# What makes an antigen a food allergen?

**DOI:** 10.1186/2045-7022-1-S1-S2

**Published:** 2011-08-12

**Authors:** Clare Mills

**Affiliations:** 1 Institute of Food Research, Norwich, UK

## 

A limited number of foods are responsible for the majority of reactions in IgE-mediated food allergy, seafood, peanut and tree nut allergies predominating in adults whilst cow’s milk and hen’s egg are important allergenic foods for infants. Over the past 10 years there has been an explosion in the numbers of well characterized allergens which have been sequenced and collected into a number of databases which has facilitated bioinformatic analyses and allowed food allergens to be classified according to their structural and biological properties. This has shown that food allergens belong to only a limited number of protein superfamilies, with animal and plant derived food allergens showing similar distributions, the majority of allergens in each group falling into just three to four families with a tail of between 14 to 23 families comprising between 1-3 allergens each. Thus, around 65% of plant food allergens belong to just four protein families, the prolamin, cupin, Bet v 1-like, and profilin families whilst animal food allergens can be classified into three main families, the tropomyosins, EF-hand proteins, and caseins.Such patterns of behavior beg the questions what makes some foods such as peanut so much more allergenic than other closely related foods such as pea? Why do certain protein scaffolds dominate the landscape of allergen structures? Can we identify structural features that predispose certain proteins to becoming allergens?

That the relationship between protein structure and allergenicity is not straight forward is indicated by the fact that previous detailed analysis of the secondary structure elements in proteins has not shown any relationship with allergenicity. It is further complicated by factors such as food processing and modification of allergen structures during digestion. Only a small number of the total number of proteins expressed in, for example an edible seed such as peanuts, have been defined as allergens and abundance alone does not account for the allergenic potency of certain proteins. Another factor that may be involved in determining allergenic potential of certain proteins is their stability to conditions commonly used in processing foods, especially in food such as peanuts which are rarely consumed raw. Lastly, in order for food proteins to either sensitize or elicit an allergic reaction they must also be bioaccessible, i.e. released from the food matrix and then survive gastrointestinal processing to be presented to the mucosal immune system in an immunologically relevant form. Structural therefore factors may contribute to the allergenicity of certain protein scaffolds and may vary between different scaffolds. Future work will focus on how the food matrix may modulate the bioaccessibility and digestion properties of these proteins and the route by which specific proteins drive class switching in B cells. Only by combining the knowledge and skills of allergen biochemists, immunologists and clinical allergists will the step-changes in thinking be achieved to address these questions in future.

